# Anhedonia across borders: Transdiagnostic relevance of reward dysfunction for noninvasive brain stimulation endophenotypes

**DOI:** 10.1111/cns.13230

**Published:** 2019-10-22

**Authors:** Maria Chiara Spano, Marco Lorusso, Mauro Pettorruso, Francesca Zoratto, Daniela Di Giuda, Giovanni Martinotti, Massimo di Giannantonio

**Affiliations:** ^1^ Department of Neuroscience, Imaging and Clinical Sciences University “G. d’Annunzio” of Chieti‐Pescara Chieti Italy; ^2^ Reference Centre for Behavioural Sciences and Mental Health Istituto Superiore di Sanità Rome Italy; ^3^ Fondazione Policlinico Universitario A. Gemelli IRCCS Roma Italy; ^4^ Università Cattolica del Sacro Cuore Roma Italy; ^5^ Department of Pharmacy, Pharmacology and Clinical Science University of Hertfordshire Herts UK

**Keywords:** addiction, depression, hedonic tone dysfunction, neuromodulation, schizophrenia, SHAPS, transcranial direct current stimulation, transcranial magnetic stimulation

## Abstract

**Introduction:**

Anhedonia is a transdiagnostic psychopathological dimension, consisting in the impaired ability to experience pleasure. In order to further our understanding of its neural correlates and to explore its potential relevance as a predictor of treatment response, in this article we systematically reviewed studies involving anhedonia and neuromodulation interventions, across different disorders.

**Methods:**

We included seven studies fulfilling inclusion/exclusion criteria and involving different measures of anticipatory and consummatory anhedonia, as well as different noninvasive brain stimulation interventions (transcranial magnetic stimulation and transcranial direct current stimulation). Studies not exploring hedonic measures or not involving neuromodulation intervention were excluded.

**Results:**

All the included studies entailed the use of rTMS protocols in one of the diverse prefrontal targets. The limited amount of studies and the heterogeneity of stimulation protocols did not allow to draw any conclusion with regard to the efficacy of rTMS in the treatment of transnosographic anhedonia. A potential for anhedonia in dissecting possible endophenotypes of different psychopathological conditions preliminarily emerged.

**Conclusions:**

Anhedonia is an underexplored condition in neuromodulation trials. It may represent a valuable transdiagnostic dimension that requires further examination in order to discover new clinical predictors for treatment response.

## 1 BACKGROUND

Transdiagnostic psychopathological dimensions have been increasingly recognized as relevant factors in the forecast of a more accurate classification and treatment of mental disorders.^1^ The Research Domain Criteria (RDoC) strategic plan defines these markers as continuous dimensions increasingly present from general population to, in higher extent, the pathophysiology of mental conditions.^2^ Moreover, transdiagnostic dimensions could be relevant in the clustering of mental disorders, in order to define different pathophysiologically based disease subtypes and possible predictors of treatment outcome.^3^ Growing research is investigating possible biotypes of mental disorders,^4^ with a predictive potential in terms of treatment response.

Anhedonia is a relevant, and often under‐considered, transdiagnostic psychopathological dimension.^5^ It is defined as the inability to experience pleasure or interest in almost all activities of daily life.^6^ There are two faces of anhedonia that could be analyzed: the consummatory and anticipatory anhedonia. The consummatory pleasure consists in the immediate satisfaction and pleasurable feelings that are linked to the realization of a desire. On the other hand, the anticipatory pleasure is the association to the expectation of a pleasurable reward and therefore is connected to motivation.^7^

Recently, neuromodulation interventions—also called noninvasive brain stimulation (NIBS) —have reached greater attention as tools for modulating local bran activity and as treatment options in several disorders.^8^ Repetitive transcranial magnetic stimulation (rTMS) and transcranial direct current stimulation (tDCS) are noninvasive and safe techniques that could inhibit or promote local neural activity in the underlying cortical areas. Also, following the neuronal projections of the targeted areas, it has been hypothesized that there could be a wider‐range action,^9^ while is not clear if in its mechanism of action, a modulation of the synaptic plasticity could be involved.^10^ The application of NIBS in different conditions has been found to determine a clinical response in a portion, but not all, of the treated patients.^11^ Nowadays, the efforts in medical research to improve and promote the neuromodulation interventions are focused on detecting different endophenotypes of mental diseases that could be more predictive of a better response to treatment.

Neuromodulation interventions have been tested in several conditions involving anhedonia by using different stimulation protocols and targeted areas, most leading to mixed results.^12^, ^13^, ^14^ Interestingly, in pharmacological studies of MDD samples, the occurrence of anhedonia has been found to be predictive of poor treatment response, limited recovery and lower quality of life.^15^ Considered the potential relevance of anhedonia in differentially affecting the clinical course of involved conditions, in this paper we aimed at exploring the concept of anhedonia as a putative transdiagnostic dimension characterizing different disorders’ endophenotypes. In order to preliminarily test the hypothesis that reward dysfunctions could be proposed as a predictor of NIBS treatment response, we systematically reviewed original articles both involving the application of neuromodulation interventions (ie, rTMS and tDCS) and focusing on psychometric scales and cognitive tasks measuring anhedonia and reward dysfunctions.

### 1.1 Transdiagnostic relevance of anhedonia in psychopathology

Anhedonia is a core symptom of major depressive disorder (MDD; especially in the definition of melancholic subtype). It is commonly found in the mental health history of patients who attempted suicide and have succeeded; anhedonic patients have greater social impairment, higher levels of hopelessness and are usually younger than nonanhedonic patients. 16, 17 Interestingly, anhedonia has been found in bipolar disorder also during euthymic phases,^18^ and it has been suggested as vulnerability factor involved in comorbid bipolar conditions.^19^

Anhedonia is a relevant negative symptom in schizophrenia (SZ)^20^ and social anhedonia, defined as the lack of pleasure or reward from social situations, is a key aspect of schizotypal personality disorder.^21^

Moreover, anhedonia is a relevant symptom in patients with substance use disorders (SUDs), since it can represent a symptom of abstinence and therefore it may predict relapse,^22^, ^23^, ^24^ posttraumatic stress disorder (PTSD),^25^, ^26^ anxiety disorder,^27^ and obsessive‐compulsive disorder.^7^ Nonetheless, anhedonia is not only a symptom of various psychiatric disorders but is also often present in other conditions such as Parkinson's disease,^28^, ^29^ over‐eating or risky behaviors.^30^, ^31^, ^32^ According to a recent review,^33^ anhedonia appears to be a heritable trait with both a biological and clinical basis and an anhedonic endophenotype could possibly be identified. Anhedonia is a trait linked to many mental diseases, and it represents a potential marker of vulnerability, especially for depression. The biological hypothesis could be that anhedonia is a symptom connected to a dysfunctional mechanism between life stressors and the brain reward system.^34^

Recent evidence indicates the presence of possible overlapping in the neural substrates of behavioral and cognitive processing in MDD and SZ conditions sharing marked anhedonia.^35^, ^36^ Although the transnosographic nature of anhedonia is well‐established, the neurobiological underpinnings of hedonic dysfunctions are still unclear,^37^ as well as it is uncertain if the neurobiological mechanisms are the same in the different mental diseases.

Animal models of anhedonia are valuable tools allowing to explore the neurobiological underpinnings as well as to search for possible predictors of treatment outcome. Data from rodent models are starting to provide important insights into the pathophysiology of anhedonia,^38^ especially through models impairing the responses to rewarding stimuli (eg, sucrose preference) or through the use of dopamine‐related transgenic approaches.^39^

### 1.2 Anhedonia and pharmacological treatments: outcome measure or predictor of response?

Since anhedonia is a difficult‐to‐treat target, several therapeutic approaches have been proposed, including psychosocial interventions, antipsychotics (for SZ conditions^40^), antidepressants (in mood disorders^41^), and neuromodulation interventions (in addictive use disorders^12^, ^42^).

The hypothesis that anhedonia is due to a dopaminergic hypofunction of the reward system leads to the implementation of pharmacological therapeutic approaches that are based on drugs that modulate that neurotransmitter as well as psychostimulants, dopamine agonists and norepinephrine or dopamine reuptake inhibitor such as Bupropion.^23^, ^43^, ^44^ Since, as mentioned before, anhedonia is a well‐known symptom in drugs’ withdrawal, many efforts are made in finding a symptomatic treatment for patients with substance use disorder. A recent study proved the efficacy on the melancholic trait of acetyl‐l‐carnitine (ALC) on alcoholic patients with anhedonic features.^45^ Recent evidence on this field highlighted the efficacy of second‐generation antipsychotics on anhedonic traits, like quetiapine and aripiprazole.^46^, ^47^, ^48^ Hence, such therapies could be also useful in dual diagnosis patients with anhedonia.^49^

Only a few studies investigated the effect of antidepressants on anhedonia. A recent review^50^ analyzed the possible effect of agomelatine. Its action appears to be mediated mainly through neurotrophins elevation,^51^ but further studies are required to prove its efficacy in reducing anhedonic symptoms.^50^

Preliminary studies on rats showed that while fluoxetine did not show any antianhedonic properties, imipramine did, but only on a subgroup. In this trial, the researchers have also studied the effect of other drugs, understanding that while clozapine and lithium showed an antianhedonic effect, haloperidol did not equivalently. Such findings confirm that dopaminergic dysfunction has a relevant role in anhedonia; drugs that interact with the dopaminergic system could be utilized to treat anhedonia and therefore the underlying mental illness.^52^

Finally, a recent systematic review by Cao and colleagues^53^ analyzed 17 studies on MDD patients with anhedonic features treated with 14 different antidepressants. This review suggested that antidepressants improve anhedonic symptoms, yet no significant difference was found among subjects.

Another valid therapeutic approach could be psychotherapy. Behavioral activation (BA), which was initially developed as part of cognitive behavioral therapy (CBT), seems to be useful in cases with anticipatory anhedonia. Such results seem to be confirmed by fMRI studies.^54^

## 2 MATERIALS AND METHODS

A literature research of the databases PubMed/MEDLINE, ISI Web of Science, and Scopus was conducted in order to find appropriate published articles on the application of neuromodulation interventions (ie, TMS and tDCS) in any clinical and nonclinical sample in which anhedonic symptoms were assessed.

A search algorithm was employed based on appropriate combinations of the terms: “anhedonia”, “pleasure”, “SHAPS”, “hedonic”, “neuromodulation”, “noninvasive brain stimulation”, “transcranial magnetic stimulation”, “repetitive “transcranial magnetic stimulation”, “direct transcranial current stimulation”, “tDCS”, “TMS”, “rTMS”.

The search was conducted on December 14, 2018. It included all publications prior to December 2018; no earliest date limit was applied. To expand the search, reference lists of the retrieved articles were also screened for additional studies.

The search strategy yielded 22 records. All original articles [open‐label trials (OLTs) or double‐blind trials, prospective or retrospective observational studies, case reports, and letter to editor], written in English language, were eligible for inclusion. We excluded studies focusing on animal models, reviews, commentaries, meta‐analysis, studies not involving any neuromodulation intervention, and studies not analyzing data referred to anhedonic measures.

**Figure 1:**
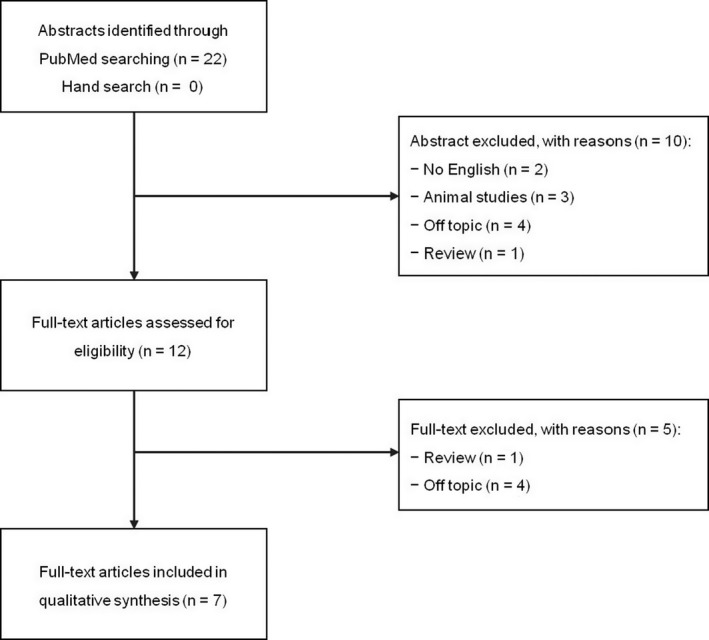
PRISMA flow diagram of systematic review

Two researchers (MCS and ML) independently reviewed the titles and abstracts of the retrieved articles, applying the above‐mentioned inclusion and exclusion criteria. By reading titles and abstracts, 10 records were excluded. The researchers then independently reviewed the full‐text version of the articles to confirm their eligibility for inclusion, or subsequent exclusion. Seven out of 12 studies fulfilled the above‐mentioned criteria and were therefore included in the qualitative synthesis (Figure 1).

For each study selected for inclusion, details regarding the publication were gathered (ie, author names, year of publication, and country of origin); data were collected on the diagnosis, on neuromodulation interventions and protocols applied (type, targeted area, and number of sessions), as well as on methods used for the assessment of anhedonia on the main findings concerning hedonic functions reported in the study. All of this information was extracted by one author and subsequently verified, independently, by the other.

## 3 RESULTS

The qualitative synthesis included seven papers (Table 1), for a total number of 201 subjects (161 males and 40 females) with 83 healthy controls and 118 patients affected by: schizophrenia (SZ; N = 45), treatment‐resistant depression (TRD; N = 58), cocaine use disorder (CUD; N = 15). Concerning the design, the studies included were as follows: one randomized controlled trial (RCT) or sham‐controlled intervention; five open‐label studies(OL); and one study with a crossover design.

**Table 1:** Summary of included studies

Authors	Study design	Sample, Diagnosis	Stimulation intervention	Targeted area	No. of Sessions	Assessment	Selected results
Downar et al (2016)	OL	47 MDE: (38 *MDD, *9 *BD*)	rTMS	DMPFC	20	HAM‐D‐17, BDI‐II, Beck Anxiety Inventory, 16‐item self‐rated Quick Inventory of Depressive Symptomatology (QIDS), Sheehan Disability Scale, Quality of Life Enjoyment and Satisfaction Questionnaire, Warwick‐Edinburgh Mental Well‐Being Scale	Nonresponders had a higher baseline anhedonia symptomatology.
Duprat et al (2016)	CO	22 HC	iTBS	Left DLPFC	1‐1	Probabilistic Learnig Task, TEPS	The more hedonic the subjects (regarding consummatory aspects) is, the more iTBS could modulate reward system (increasing dopamine release).
Hurlermann et al (2015)	OL	41 HC	iTBS	DMPFC DLPFC	2	fMRI localizer task, Cognitive emotion judgment task, emotion‐modulated startle paradigm.	NS
Ulrich et al (2018)	OL	20 HC	iTBS + cTBS	Right‐medial VLPFC	1‐1	FCQ‐S	Modulation of processes of revaluation of hedonic food stimuli driven by TMS on rVLPFC. “Reshape” of hedonic food regulation.
Prikryl et al (2013)	RCT	45 SZ	rTMS	Left PFC	15	SANS, SAPS, MADRS, CDSS	Significant improvement in anhedonia subscore (*P* < .001).
Russo et al (2018)	OL	11 TRD	TMS	Left DLPFC	36	PES, IDS‐SR, PHQ‐9, SHAPS	Not significant improvement in SHAPS score.
Pettorruso et al (2018)	OL	15 CUD	rTMS	Left DLPFC	10	TEPS, VAS, CSSA, UDS.	Improvements in anhedonia symptoms, more pronounced in high‐craving CUD subjects.

Abbreviations: BDI‐II, Beck Depression Inventory; CDSS, Calgary Depression Scale for Schizophrenia; CO, crossover study; CSSA, Cocaine Selective Severity Assessment; cTBS, continuous theta‐burst stimulation; DLPFC, dorsolateral prefrontal cortex; DMPFC, dorsomedial prefrontal cortex; FCQ‐S, Food Craving Questionnaire‐Short; fMRI, functional magnetic resonance; HAM‐D‐17, Hamilton Depression Scale 17 items; IDS‐SR, Inventory of Depressive Symptomatology (Self‐Report); iTBS, intermittent theta‐burst stimulation; MADRS, The Montgomery‐Åsberg Depression Rating Scale; OL, open‐label study; PES, Pleasant Event Schedule; PHQ‐9, Patient Health Questionnaire‐9; RCT, randomized controlled trial; rTMS, repetitive transcranial magnetic stimulation; SANS, Scale for the Assessment of Negative Symptoms; SAPS, Scale for the Assessment of Positive Symptoms; SHAPS, Snaith‐Hamilton Pleasure Scale; TEPS, Temporal Experience of Pleasure Scale; UDS, urine drug screen; VAS, Visual Analogue Scale; VLPFC, ventromedial prefrontal cortex.

### 3.1 Depression

Downar and colleagues,^55^ in an open‐label study, recruited 47 patients (20 males and 27 females) with unipolar (n = 38) and bipolar (n = 9) TRD. These patients were treated with an add‐on high‐frequency rTMS over the dorsomedial prefrontal cortex (DMPFC), for a total of 20 daily sessions. Psychometric instruments showed a response rate in almost one‐half of the sample (50% reduction in symptoms' severity). They reported that nonrespondent patients were more likely to be anhedonic at the baseline. Furthermore, nonresponders showed significant lower connectivity through the reward pathway on baseline functional magnetic resonance.

Russo and colleagues^14^ explored the effects of a standard course of rTMS (5 daily sessions per week, up to 36 sessions) in an open‐design involving 11 female outpatients with a diagnosis of treatment‐resistant depression (TRD). From the second week of treatment, patients also received the behavioral activity therapy before the rTMS session. Six patients (55%) improved after rTMS treatment, along with a nonsignificant reduction in SHAPS scores.

### 3.2 Schizophrenia

Prikryl and colleagues^56^ conducted a 3‐week double‐blind RCT involving 45 schizophrenic male patients. The study protocol established five rTMS sessions per week for three weeks, added on the current pharmacological regimen and targeting the left PFC (tangential to the midline). Twenty‐five patients received the active stimulation, 20 the sham. A significant reduction of negative and affective symptoms severity was observed, particularly in the anhedonia subscale of SANS, both in the active and sham groups.

### 3.3 Substance use disorders

Our research group, in an open‐label pilot study,^12^ explored the effects of high‐frequency rTMS (10 sessions, two daily for five consecutive days) over the left DLPFC on anhedonic symptoms, in a sample of 15 treatment‐seeking subjects with cocaine use disorder (CUD). After the neuromodulation intervention, significant changes in Temporal Experience of Pleasure Scale (TEPS) scores were observed, indicative of reduction in both anticipatory and consummatory anhedonic symptoms. Furthermore, the results indicate an inverse correlation between craving reduction and improvements in anhedonia, suggesting a potential pathophysiological link between these clinical phenomena.

### 3.4 Healthy subjects

Duprat and colleagues^57^ conducted a crossover study by using an intermittent theta‐burst stimulation (iTBS) protocol in 22 male healthy subjects. All subjects received a single active and sham stimulation over the left DLPFC, with one‐week washout period. Active stimulation was able to influence the performance in a reward‐related task, with a faster action of iTBS in subjects with higher hedonic capacity to elicit the development of a response bias. Consistently with these results, the authors argued a more pronounced sensitivity to rewarding stimuli after the neuromodulation intervention, putatively related to increased dopamine release.

In a randomized controlled trial, Hurlermann and colleagues^58^ applied two sessions (targeting DMPFC and DLPFC areas) of continuous TBS (an inhibitory NIBS protocol) in a sample of 41 male healthy subjects. Both the inhibition of left DLPFC and DMPFC resulted in the attenuation of emotional responses to positive stimuli.

Recently, in an open‐label study,^59^ 20 healthy males were recruited to perform a fMRI food/nonfood discrimination task before and after TBS. In particular, 10 patients started with iTBS followed by continuous TBS (cTBS) and vice‐versa. Both stimulation protocols targeted the right mid‐VLPFC at a 70% of RMT intensity. The two neuromodulation interventions were able to increase fMRI neural responses for low‐calorie food images. In addition, cTBS determined a significant decrease of the ventral tegmental area fMRI activation for high‐calorie foods.

## 4 DISCUSSION

Our revision of the literature highlighted only seven studies that have analyzed neuromodulation in correlation to the hedonic trait. The number of these studies is very limited, considering the constant spread of the NIBS worldwide and specifically in psychiatry. Moreover, the protocols used in the clinical trials were found to be very heterogeneous in terms of number of applications, targeted area, and study design. Anhedonia, as transnosographic symptom, has a good potential in dissecting the population involved in neuromodulation trials, in particular it could be used as an outcome predictor. As of today, only one study suggests that connectivity patterns in reward circuits and anhedonic symptoms could predict a negative answer to HF rTMS on DMPFC. On the other hand, HF rTMS seems to be efficacious in improving anhedonic function in nondepressed subjects (ie, in healthy and SUDs subjects) when applied on the left DLPFC, along with a modulation of the connected subcortical areas.^12^, ^60^, ^61^

All the included studies targeted a prefrontal area (mainly, the DLPFC and the DMPFC). Recently, different neurobiological correlates have been proposed for anticipatory and consummatory anhedonia.^20^ While consummatory anhedonia has been suggested as being mainly related to impaired ventral basal ganglia activation, the anticipatory component seems to be underpinned by frontal‐striatal networks involving dorsal anterior cingulate cortex and the middle/medial frontal gyrus. Interestingly, in a 1188 MDD patients study,^4^ four relevant biotypes based on fMRI dysfunctional connectivity patterns were identified. Two of these were found involving alterations in frontostriatal and orbitofrontal area, with significant correlations with anhedonia and psychomotor retardation. In addition, evidence highlighted an important contribution of the DMPFC to the reward value in the intertemporal choice. Furthermore, the stimulation of DMPFC was demonstrated to modulate striatal dopamine levels,^62^, ^63^ also involved in reward processing.^64^

The vast majority of clinical trials testing the efficacy of high‐frequency rTMS in patients with depressive disorders targeted the left dorsolateral prefrontal cortex, but some preliminary evidence demonstrated the efficacy of targeting different areas (ie, DMPFC).^8^ Possibly, dissecting endophenotypes based on hedonic functioning may allow the individuation of patients’ profiles differentially responding to diverse interventions. As our review shows, only a little part of clinical studies analyzed the anhedonic dimension. It would be important to better characterize clinical samples in the manner of pharmacological studies.^65^, ^66^ In fact, the occurrence of anhedonic symptoms would detect possible response predictors to neuromodulation treatment.^51^

As discussed, anhedonia has a relevant impact on patients’ quality of life. It worsens the rate of suicidal ideation in different samples^67^, ^68^, ^69^, ^70^, ^71^ and determines poor social functioning^72^ and higher and prolonged hospitalization.^73^

Lastly, anhedonia could be considered a pivotal psychopathological symptom, also noted in the DSM‐5 as a core symptom of MDD. Since it also correlates to more severe clinical frames and worse outcomes,^74^ it could be an important target for treatment. Treating anhedonia with specific tools could also improve the clinical course of these patients.

In conclusion, current literature does not support a specific role of neuromodulation interventions for the treatment of anhedonia. Nevertheless, it could be promising to dissect the involved endophenotypes to predict neuromodulation treatment response, but this proposal needs more research and verification in experimental conditions. Further studies are necessary to clarify the role of these approaches to reverse anhedonia and its disabling consequences.
